# Butane-1,2,3,4-tetra­carb­oxy­lic acid–1,10-phenanthroline–water (1/2/2)

**DOI:** 10.1107/S1600536811021398

**Published:** 2011-06-11

**Authors:** Hong-lin Zhu

**Affiliations:** a Center of Applied Solid State Chemistry Research, Ningbo University, Ningbo, Zhejiang 315211, People’s Republic of China

## Abstract

The asymmetric unit of the title compound, 2C_12_H_8_N_2_·C_8_H_10_O_8_·2H_2_O, contains one 1,10-phenanthroline mol­ecule, one half-mol­ecule of butane-1,2,3,4-tetra­carb­oxy­lic acid (H_4_BTC) and a water mol­ecule, with the complete tetra-acid generated by crystallographic inversion symmetry. Inter­molecular O—H⋯O hydrogen bonds and π–π stacking inter­actions [centroid–centroid distances = 3.672 (2) and 3.708 (2) Å form an extensive three-dimensional network, which consolidates the crystal packing.

## Related literature

For the use of H_4_BTC as a ligand in metal–organic coordination complexes, see: Delgado *et al.* (2007 ▶); Liu *et al.* (2008 ▶); Xu *et al.* (2010 ▶); Zhu *et al.* (2011 ▶). For co-crystals involving H_4_BTC, see: Cheng *et al.* (2009 ▶); Najafpour *et al.* (2008 ▶). For details of the Cambridge Structural Database, see: Allen  (2002 ▶).
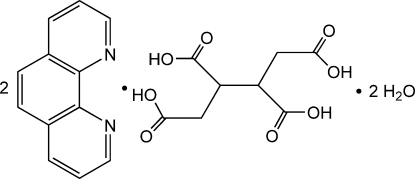


## Experimental

### 

#### Crystal data

2C_12_H_8_N_2_·C_8_H_10_O_8_·2H_2_O
*M* *_r_* = 630.60
Triclinic, 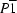

*a* = 7.9472 (16) Å
*b* = 9.884 (2) Å
*c* = 10.628 (2) Å
α = 84.37 (3)°
β = 70.12 (3)°
γ = 72.72 (3)°
*V* = 749.7 (3) Å^3^
*Z* = 1
Mo *K*α radiation
μ = 0.11 mm^−1^
*T* = 293 K
0.58 × 0.34 × 0.10 mm

#### Data collection

Rigaku R-AXIS RAPID diffractometer
Absorption correction: multi-scan (*ABSCOR*; Higashi, 1995 ▶) *T*_min_ = 0.950, *T*_max_ = 0.990
7400 measured reflections
3396 independent reflections
1960 reflections with *I* > 2σ(*I*)
*R*_int_ = 0.024

#### Refinement

*R*[*F* ^2^ > 2σ(*F* ^2^)] = 0.048
*wR*(*F* ^2^) = 0.188
*S* = 1.17
3396 reflections
208 parameters
H-atom parameters constrained
Δρ_max_ = 0.30 e Å^−3^
Δρ_min_ = −0.34 e Å^−3^

### 

Data collection: *RAPID-AUTO* (Rigaku, 1998 ▶); cell refinement: *RAPID-AUTO*; data reduction: *CrystalStructure* (Rigaku/MSC, 2004 ▶); program(s) used to solve structure: *SHELXS97* (Sheldrick, 2008 ▶); program(s) used to refine structure: *SHELXL97* (Sheldrick, 2008 ▶); molecular graphics: *SHELXTL* (Sheldrick, 2008 ▶); software used to prepare material for publication: *SHELXL97*.

## Supplementary Material

Crystal structure: contains datablock(s) global, I. DOI: 10.1107/S1600536811021398/sj5157sup1.cif

Structure factors: contains datablock(s) I. DOI: 10.1107/S1600536811021398/sj5157Isup2.hkl

Additional supplementary materials:  crystallographic information; 3D view; checkCIF report

## Figures and Tables

**Table 1:** Hydrogen-bond geometry (Å, °)

*D*—H⋯*A*	*D*—H	H⋯*A*	*D*⋯*A*	*D*—H⋯*A*
O1—H1*A*⋯O5^iii^	0.87	1.70	2.565 (3)	172
O4—H4*A*⋯N2	0.86	1.90	2.723 (3)	159
O5—H5*B*⋯O2^iv^	0.88	1.98	2.817 (3)	160
O5—H5*C*⋯N1	0.85	2.09	2.858 (3)	149

Symmetry codes: (iii) 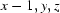; (iv) 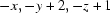.

**Fig. 1.**
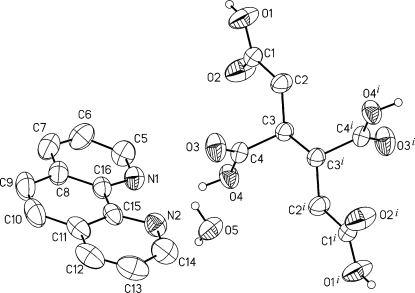
The content of asymmetric unit showing the atomic numbering and 45% probability displacement ellipsoids [Symmetry codes: (i) -x, -y + 1, -z + 1].

**Fig. 2.**
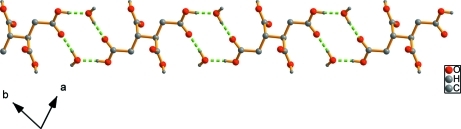
Supramolecular assembly of a one-dimensional chain via O–H···O hydrogen bonds.

**Fig. 3.**
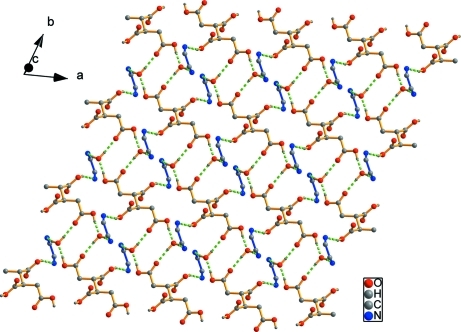
Supramolecular assembly of a two-dimensional layer via O–H···N hydrogen bonds.

**Fig. 4.**
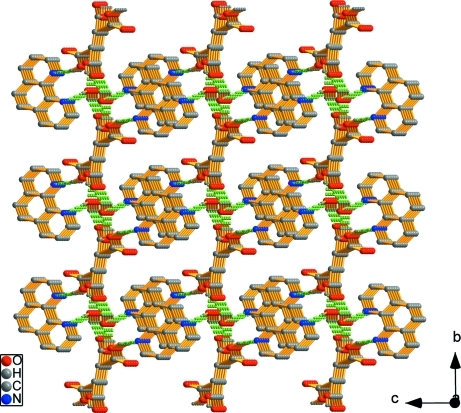
Supramolecular assembly of three-dimensional architecture through π–π stacking interactions.

## supplementary crystallographic information

### Comment

Systems with butane-1,2,3,4-tetracarboxylic acid (H_4_BTC) as a ligand have been widely studied (Delgado *et al.*, 2007; Liu *et al.*, 2008; Xu *et al.*, 2010; Zhu *et al.*, 2011). A search of the Cambridge structural database (version 5.32, May 2011) (Allen, 2002) showed that most of the literature dealing with butane-1,2,3,4-tetracarboxylic acid concentrated on metal-organic coordination complexes. In contrast, utilization of the butane-1,2,3,4-tetracarboxylic acid seems relatively limited in the construction of co-crystals (Cheng *et al.*, 2009; Najafpour *et al.*, 2008). In this paper, we report the structure of the title cocrystal.

The asymmetric unit of the title cocrystal consists of one 1,10-phenanthroline, unit one half molecule of butane-1,2,3,4-tetracarboxylic acid and a water molecule as depicted in Figure 1. The present 1,10-phenanthroline molecule preserves a nearly perfect coplanarity with a maximum deviation from the best fit meanplane 0.123 (1) Å. The carboxylato group with C1 and C4 atoms is gauche with the C1–C2–C3–C4 torsion angle being 63.35 (2)°. These values agree well with reported structures (Cheng *et al.*, 2009; Najafpour *et al.*, 2008;). The butane-1,2,3,4-tetracarboxylic acid molecules and water molecules are interlinked *via* O–H···O hydrogen bonds to generate a 1-dimensional supramolecular chain (Figure 2), which is further interconnected by interchain O–H···N hydrogen bonds to construct a 2-dimensional layer parallel to the (001) plane (Figure 3). The resulting layers are arranged in such a way that the 1,10-phenanthroline ligands are each sandwiched between two antiparallel phen neighbors from different adjacent layers, and the mean interplanar distances between the neighboring phen ligands are 3.67 Å and 3.71 Å, suggesting significant intermolecular face-to-face π–π stacking interactions. Such interlayer interactions are regarded as the driving forces to assemble the layers into a three-dimensional supramolecular architecture as shown in Figure 4.

### Experimental

All chemicals were obtained from commerical sources and were used as obtained. 1,10-phenanthroline (0.1983 g, 1.00 mmol) was added to a stirred mixture solution of butane-1,2,3,4-tetracarboxylic acid (0.1173 g, 0.50 mmol) in 10 ml H_2_O and 10 ml me thanol, and the resulting mixture was stirred for 30 min. Colorless crystals were obtained from the solution after standing at room temperature for two months.

### Refinement

H atoms bonded to C atoms were placed in geometrically calculated positions and were refined using a riding model, with *U*_iso_(H) = 1.2 *U*_eq_(C). H atoms attached to O atoms were found in a difference Fourier synthesis and were refined using a riding model, with the O–H distances fixed as initially found and with *U*_iso_(H) values set at 1.2 *U*_eq_(O).

### Crystal data

**Table:**

2C_12_H_8_N_2_·C_8_H_10_O_8_·2H_2_O	*Z* = 1
*M**_r_* = 630.60	*F*(000) = 330
Triclinic, *P*1	*D*_x_ = 1.397 Mg m^−^^3^
Hall symbol: -P 1	Mo *K*α radiation, λ = 0.71073 Å
*a* = 7.9472 (16) Å	Cell parameters from 4546 reflections
*b* = 9.884 (2) Å	θ = 3.1–27.5°
*c* = 10.628 (2) Å	µ = 0.11 mm^−^^1^
α = 84.37 (3)°	*T* = 293 K
β = 70.12 (3)°	Block, colorless
γ = 72.72 (3)°	0.58 × 0.34 × 0.10 mm
*V* = 749.7 (3) Å^3^	

### Data collection

**Table:**

Rigaku R-AXIS RAPID diffractometer	3396 independent reflections
Radiation source: fine-focus sealed tube	1960 reflections with *I* > 2σ(*I*)
graphite	*R*_int_ = 0.024
ω scans	θ_max_ = 27.5°, θ_min_ = 3.1°
Absorption correction: multi-scan (*ABSCOR*; Higashi, 1995)	*h* = −10→10
*T*_min_ = 0.950, *T*_max_ = 0.990	*k* = −12→12
7400 measured reflections	*l* = −13→13

### Refinement

**Table:**

Refinement on *F*^2^	Primary atom site location: structure-invariant direct methods
Least-squares matrix: full	Secondary atom site location: difference Fourier map
*R*[*F*^2^ > 2σ(*F*^2^)] = 0.048	Hydrogen site location: inferred from neighbouring sites
*wR*(*F*^2^) = 0.188	H-atom parameters constrained
*S* = 1.17	*w* = 1/[σ^2^(*F*_o_^2^) + (0.0607*P*)^2^ + 0.4694*P*] where *P* = (*F*_o_^2^ + 2*F*_c_^2^)/3
3396 reflections	(Δ/σ)_max_ < 0.001
208 parameters	Δρ_max_ = 0.30 e Å^−^^3^
0 restraints	Δρ_min_ = −0.34 e Å^−^^3^

### Special details

**Table:**

Geometry. All e.s.d.'s (except the e.s.d. in the dihedral angle between two l.s. planes) are estimated using the full covariance matrix. The cell e.s.d.'s are taken into account individually in the estimation of e.s.d.'s in distances, angles and torsion angles; correlations between e.s.d.'s in cell parameters are only used when they are defined by crystal symmetry. An approximate (isotropic) treatment of cell e.s.d.'s is used for estimating e.s.d.'s involving l.s. planes.
Refinement. Refinement of *F*^2^ against ALL reflections. The weighted *R*-factor *wR* and goodness of fit *S* are based on *F*^2^, conventional *R*-factors *R* are based on *F*, with *F* set to zero for negative *F*^2^. The threshold expression of *F*^2^ > σ(*F*^2^) is used only for calculating *R*-factors(gt) *etc*. and is not relevant to the choice of reflections for refinement. *R*-factors based on *F*^2^ are statistically about twice as large as those based on *F*, and *R*- factors based on ALL data will be even larger.

### Fractional atomic coordinates and isotropic or equivalent isotropic displacement parameters (Å^2^)

**Table:**

	*x*	*y*	*z*	*U*_iso_*/*U*_eq_	
O1	−0.5184 (3)	0.7050 (2)	0.4404 (3)	0.0745 (7)	
H1A	−0.5899	0.7919	0.4447	0.089*	
O2	−0.3498 (3)	0.8187 (2)	0.4902 (3)	0.0734 (7)	
C1	−0.3757 (4)	0.7102 (3)	0.4718 (3)	0.0434 (6)	
C2	−0.2475 (4)	0.5669 (3)	0.4825 (3)	0.0484 (6)	
H2A	−0.2240	0.5103	0.4055	0.058*	
H2B	−0.3086	0.5200	0.5619	0.058*	
C3	−0.0619 (3)	0.5737 (2)	0.4898 (2)	0.0389 (5)	
H3A	−0.0862	0.6351	0.5647	0.047*	
C4	0.0425 (4)	0.6356 (2)	0.3614 (2)	0.0408 (5)	
O3	0.0605 (3)	0.5952 (2)	0.25216 (19)	0.0652 (6)	
O4	0.1121 (3)	0.73303 (19)	0.37990 (18)	0.0532 (5)	
H4A	0.1639	0.7706	0.3054	0.064*	
N1	0.0963 (3)	1.0528 (2)	0.2323 (2)	0.0514 (6)	
C5	−0.0353 (5)	1.1744 (3)	0.2717 (3)	0.0613 (8)	
H5A	−0.0890	1.1934	0.3629	0.074*	
C6	−0.0969 (5)	1.2746 (3)	0.1844 (4)	0.0705 (9)	
H6A	−0.1915	1.3571	0.2170	0.085*	
C7	−0.0175 (5)	1.2505 (3)	0.0505 (3)	0.0677 (9)	
H7A	−0.0565	1.3168	−0.0096	0.081*	
C8	0.1230 (4)	1.1255 (3)	0.0040 (3)	0.0546 (7)	
C9	0.2172 (5)	1.0959 (4)	−0.1356 (3)	0.0696 (9)	
H9A	0.1810	1.1599	−0.1982	0.084*	
C10	0.3561 (6)	0.9781 (4)	−0.1774 (3)	0.0734 (10)	
H10A	0.4185	0.9633	−0.2687	0.088*	
C11	0.4106 (4)	0.8744 (3)	−0.0853 (3)	0.0571 (7)	
C12	0.5552 (5)	0.7492 (4)	−0.1252 (4)	0.0746 (10)	
H12A	0.6236	0.7328	−0.2154	0.089*	
C13	0.5961 (5)	0.6515 (4)	−0.0328 (4)	0.0777 (10)	
H13A	0.6935	0.5690	−0.0586	0.093*	
C14	0.4888 (5)	0.6777 (3)	0.1014 (4)	0.0709 (9)	
H14A	0.5149	0.6093	0.1639	0.085*	
C15	0.3153 (4)	0.8952 (3)	0.0537 (3)	0.0466 (6)	
C16	0.1741 (4)	1.0267 (3)	0.0985 (2)	0.0449 (6)	
N2	0.3519 (3)	0.7946 (2)	0.1444 (2)	0.0552 (6)	
O5	0.2570 (3)	0.9523 (2)	0.4390 (2)	0.0597 (6)	
H5B	0.2932	1.0275	0.4404	0.072*	
H5C	0.1736	0.9754	0.4011	0.072*	

### Atomic displacement parameters (Å^2^)

**Table:**

	*U*^11^	*U*^22^	*U*^33^	*U*^12^	*U*^13^	*U*^23^
O1	0.0631 (13)	0.0483 (11)	0.131 (2)	−0.0092 (10)	−0.0622 (14)	0.0059 (12)
O2	0.0697 (14)	0.0409 (11)	0.124 (2)	−0.0077 (10)	−0.0544 (14)	−0.0078 (11)
C1	0.0420 (13)	0.0434 (13)	0.0460 (14)	−0.0107 (11)	−0.0188 (11)	0.0070 (11)
C2	0.0450 (14)	0.0369 (12)	0.0677 (17)	−0.0104 (11)	−0.0269 (13)	0.0080 (12)
C3	0.0394 (12)	0.0340 (11)	0.0442 (13)	−0.0094 (10)	−0.0166 (10)	0.0046 (10)
C4	0.0475 (14)	0.0363 (12)	0.0392 (13)	−0.0102 (10)	−0.0178 (11)	0.0053 (10)
O3	0.0941 (17)	0.0659 (13)	0.0441 (11)	−0.0352 (12)	−0.0239 (11)	0.0055 (9)
O4	0.0683 (13)	0.0509 (11)	0.0502 (11)	−0.0332 (10)	−0.0205 (9)	0.0110 (8)
N1	0.0597 (14)	0.0524 (13)	0.0431 (12)	−0.0176 (11)	−0.0171 (11)	0.0022 (10)
C5	0.068 (2)	0.0575 (17)	0.0536 (17)	−0.0092 (15)	−0.0186 (15)	−0.0078 (14)
C6	0.077 (2)	0.0546 (17)	0.080 (2)	−0.0035 (16)	−0.0384 (19)	−0.0002 (16)
C7	0.082 (2)	0.0611 (19)	0.070 (2)	−0.0191 (17)	−0.0424 (19)	0.0141 (16)
C8	0.0687 (18)	0.0576 (16)	0.0503 (16)	−0.0299 (15)	−0.0282 (14)	0.0125 (13)
C9	0.093 (3)	0.083 (2)	0.0443 (16)	−0.041 (2)	−0.0266 (17)	0.0164 (16)
C10	0.091 (3)	0.096 (3)	0.0380 (15)	−0.048 (2)	−0.0102 (16)	0.0071 (17)
C11	0.0575 (17)	0.0677 (18)	0.0488 (16)	−0.0326 (15)	−0.0060 (13)	−0.0067 (14)
C12	0.066 (2)	0.086 (2)	0.066 (2)	−0.0365 (19)	0.0029 (17)	−0.0179 (19)
C13	0.058 (2)	0.064 (2)	0.098 (3)	−0.0149 (16)	−0.0048 (19)	−0.025 (2)
C14	0.068 (2)	0.0519 (17)	0.082 (2)	−0.0128 (15)	−0.0131 (18)	−0.0047 (16)
C15	0.0533 (15)	0.0488 (14)	0.0434 (14)	−0.0281 (12)	−0.0121 (12)	0.0034 (11)
C16	0.0548 (15)	0.0496 (14)	0.0382 (13)	−0.0246 (12)	−0.0179 (11)	0.0054 (11)
N2	0.0594 (15)	0.0461 (12)	0.0576 (14)	−0.0170 (11)	−0.0144 (12)	0.0017 (11)
O5	0.0634 (13)	0.0495 (11)	0.0780 (14)	−0.0128 (9)	−0.0418 (11)	0.0047 (10)

### Geometric parameters (Å, °)

**Table:**

O1—C1	1.302 (3)	C7—H7A	0.9300
O1—H1A	0.8734	C8—C16	1.406 (4)
O2—C1	1.196 (3)	C8—C9	1.431 (4)
C1—C2	1.499 (3)	C9—C10	1.333 (5)
C2—C3	1.525 (3)	C9—H9A	0.9300
C2—H2A	0.9700	C10—C11	1.423 (5)
C2—H2B	0.9700	C10—H10A	0.9300
C3—C4	1.514 (3)	C11—C12	1.399 (5)
C3—C3^i^	1.540 (4)	C11—C15	1.416 (4)
C3—H3A	0.9800	C12—C13	1.359 (5)
C4—O3	1.214 (3)	C12—H12A	0.9300
C4—O4	1.303 (3)	C13—C14	1.393 (5)
O4—H4A	0.8635	C13—H13A	0.9300
N1—C5	1.330 (4)	C14—N2	1.321 (4)
N1—C16	1.361 (3)	C14—H14A	0.9300
C5—C6	1.387 (4)	C15—N2	1.354 (4)
C5—H5A	0.9300	C15—C16	1.438 (4)
C6—C7	1.359 (5)	O5—H5B	0.8756
C6—H6A	0.9300	O5—H5C	0.8533
C7—C8	1.393 (4)		
			
Cg1···Cg3^ii^	3.672 (2)	Cg2···Cg3^iii^	3.708 (2)
			
C1—O1—H1A	106.5	C7—C8—C16	118.4 (3)
O2—C1—O1	123.2 (2)	C7—C8—C9	122.2 (3)
O2—C1—C2	123.5 (2)	C16—C8—C9	119.4 (3)
O1—C1—C2	113.4 (2)	C10—C9—C8	121.1 (3)
C1—C2—C3	112.9 (2)	C10—C9—H9A	119.5
C1—C2—H2A	109.0	C8—C9—H9A	119.5
C3—C2—H2A	109.0	C9—C10—C11	121.3 (3)
C1—C2—H2B	109.0	C9—C10—H10A	119.4
C3—C2—H2B	109.0	C11—C10—H10A	119.4
H2A—C2—H2B	107.8	C12—C11—C15	117.1 (3)
C4—C3—C2	109.7 (2)	C12—C11—C10	123.0 (3)
C4—C3—C3^i^	108.5 (2)	C15—C11—C10	119.8 (3)
C2—C3—C3^i^	112.0 (2)	C13—C12—C11	120.3 (3)
C4—C3—H3A	108.9	C13—C12—H12A	119.8
C2—C3—H3A	108.9	C11—C12—H12A	119.8
C3^i^—C3—H3A	108.9	C12—C13—C14	118.6 (3)
O3—C4—O4	124.0 (2)	C12—C13—H13A	120.7
O3—C4—C3	122.1 (2)	C14—C13—H13A	120.7
O4—C4—C3	113.9 (2)	N2—C14—C13	123.5 (4)
C4—O4—H4A	111.9	N2—C14—H14A	118.2
C5—N1—C16	117.5 (2)	C13—C14—H14A	118.2
N1—C5—C6	123.7 (3)	N2—C15—C11	122.0 (3)
N1—C5—H5A	118.1	N2—C15—C16	119.6 (2)
C6—C5—H5A	118.1	C11—C15—C16	118.5 (3)
C7—C6—C5	119.1 (3)	N1—C16—C8	121.9 (3)
C7—C6—H6A	120.4	N1—C16—C15	118.4 (2)
C5—C6—H6A	120.4	C8—C16—C15	119.7 (2)
C6—C7—C8	119.4 (3)	C14—N2—C15	118.3 (3)
C6—C7—H7A	120.3	H5B—O5—H5C	107.6
C8—C7—H7A	120.3		

Symmetry codes: (i) −*x*, −*y*+1, −*z*+1; (ii) −*x*, −*y*+2, −*z*; (iii) −*x*+1, −*y*+2, −*z*.

### Hydrogen-bond geometry (Å, °)

**Table:**

Cg1, Cg2 and Cg3 are the centroids of the N1/C5-C8/C16/, N2/C14-C11/C15, and C8-C11/C15/C16, rings respectively.

**Table:**

*D*—H···*A*	*D*—H	H···*A*	*D*···*A*	*D*—H···*A*
O1—H1A···O5^iv^	0.87	1.70	2.565 (3)	172
O4—H4A···N2	0.86	1.90	2.723 (3)	159
O5—H5B···O2^v^	0.88	1.98	2.817 (3)	160
O5—H5C···N1	0.85	2.09	2.858 (3)	149

Symmetry codes: (iv) *x*−1, *y*, *z*;  (v) −*x*, −*y*+2, −*z*+1.
